# Supplementary material for: The Glasgow Prognostic Score Predicts Survival Outcomes in Neuroendocrine Neoplasms of the Gastro–Entero–Pancreatic (GEP-NEN) System
Source: Cancers (Basel). 2022 Nov 7;14(21):5465. doi: 10.3390/cancers14215465 (PMC9656405; doi:10.3390/cancers14215465)
Supplement: Supplementary file 1 [file cancers-14-05465-s001.zip › cancers-1854742-supplementary.pdf]

Supplementary Material

Supplementary Methods

The c-index hold discriminatory power of a given statistical model (with higher values indicating superior predictive properties; ≤ 0.5 poor model, no better than predicting an outcome than random chance; 1 = perfect model, impeccable prediction of outcome according to group allocation), while the cAIC quantifies the predictive potential of statistical models upon direct comparison at low sample volumes (with lower values indicating better accuracy). A difference in cAIC values between 0 and 2 indicates the absence of significant differences in model fit while a difference between 2 and 10 suggests an increasing improvement in fit, a difference greater than 10 represents a substantial improvement in fit.

Supplementary Table S1. Data on model fit and concordance of CRP and albumin-based risk scores in GEP NEN patients.

	**OS**		**PFS**	
**Model**	**c-index**	**cAIC**	**c-index**	**cAIC**
**GPS**	0.581	1055.7	0.824	1035.10
**CAR**	0.548	1055.7	0.799	1036.74

cAIC, corrected Akaike's information criterion; CAR, CRP/albumin ratio; CI, confidence interval; GPS, Glasgow prognostic score.

Both the GPS as well as CAR were shown to hold predictive properties for both OS and PFS while the GPS was shown to be slightly superior compared to CAR. We calculated a higher c-index for the GPS and a lower value for the cAIC of GPS indicating preponderance regarding survival prediction.

Supplementary Figures

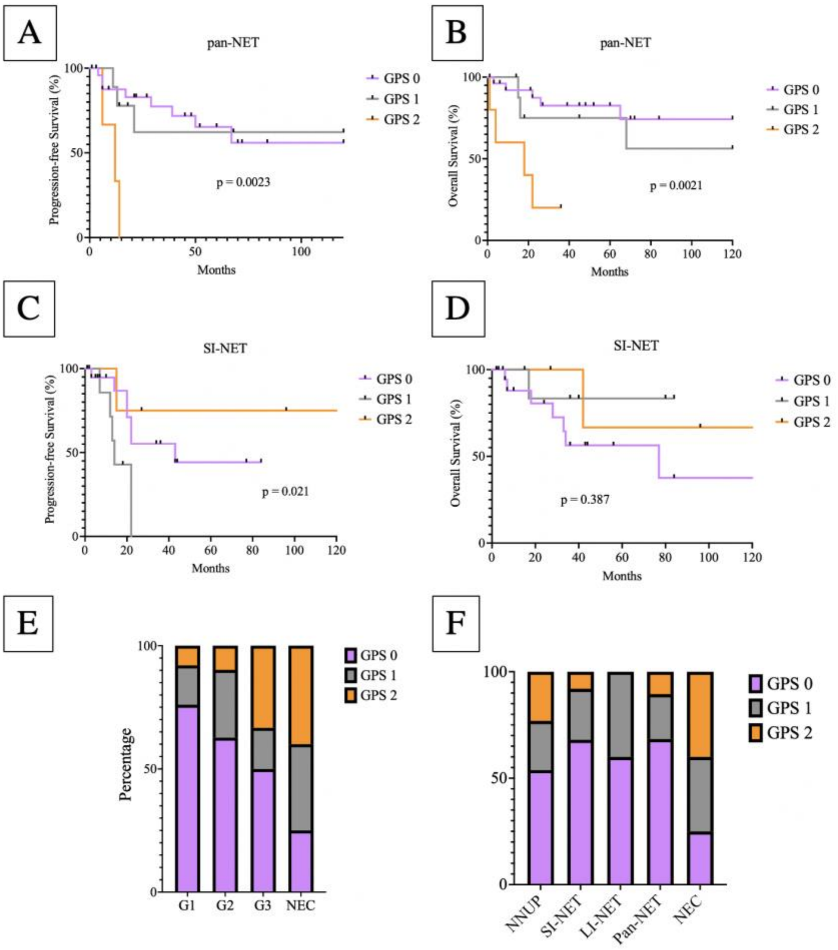

Supplementary Figure S1. PFS (**A, C**) and OS (**B, D**) in pancreatic NETs (**A, B**) and SI-NETs (**C, D**) according to GPS subgroups. **E and F** demonstrate the distribution of GPS subgroups according to histological grading (**E**) and primary tumor sites (**F**).

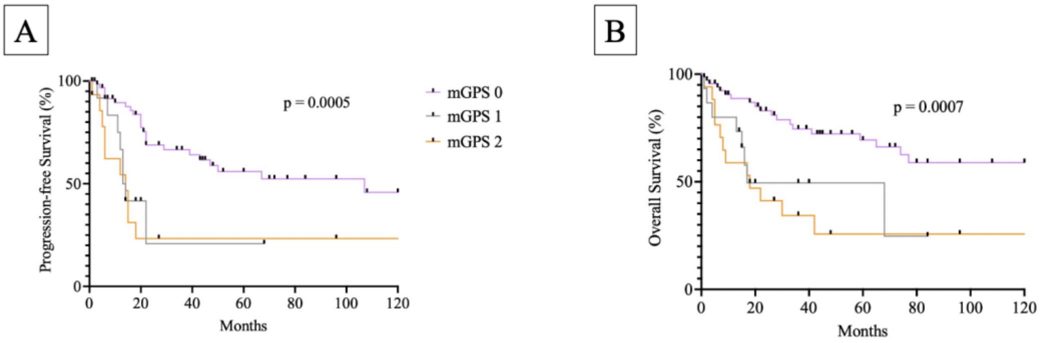

Supplementary Figure S2. Progression-free (**A**) and overall (**B**) survival according to the modified Glasgow Prognostic Score (GPS) (log-rank mGPS 0 vs. mGPS 1 vs. mGPS 2; **A, B**).

Supplementary Table S2. Second line treatment modalities of all GEP-NEN-patients included in the study.

**Characteristics**	**Overall study group (n = 35)**	**GPS 0 (n = 19)**	**GPS 1 (n = 8)**	**GPS 2 (n = 8)**
**2nd line treatment**				
Surgical resection	2	2	-	-
Chemotherapy	18	9	3	6
Targeted therapy	6	2	3	1
PRRT	8	6	2	-
Somatostatin analogues	3	2	-	1
**Best response (RECIST v1.1)**				
CR	2	2	-	-
PR	17	10	3	4
SD	12	8	2	2
PD	3	1	-	2
**Toxicity profile (NCI CTC)**				
Cytopenia grad III/IV	5	2	3	-
Emesis	3	-	1	2
Pneumonitis	-	-	-	-
Nephrotoxicity	-	-	-	-

CR, complete remission; Dfd, death from disease; GPS, Glasgow-prognostic score; NCI CTC, National Cancer Institute Common Toxicity Criteria; PD, progressive disease; PR, partial remission; PRRT, peptide-receptor-radionuclide-therapy; SD, stable disease; RECIST, Response evaluation criteria in solid tumors.

Supplementary Table S3. Third line treatment modalities of all GEP-NEN patients included in the study.

Characteristics	Overall study group (n = 13)	GPS 0 (n = 7)	GPS 1 (n = 3)	GPS 2 (n = 3)
**3rd line treatment**				
Surgical resection	-	-	-	-
Chemotherapy	10	5	2	3
Targeted therapy	1	1	-	-
PRRT	2	1	1	-
Somatostatin analogues	-	-	-	-
**Best response (RECIST v1.1)**				
CR	-	-	-	-
PR	4	2	1	1
SD	2	-	1	1
PD	7	5	1	1
**Toxicity profile (NCI CTC)**				
Cytopenia grad III/IV	2	2	-	-
Emesis	-	-	-	-
Pneumonitis	-	-	-	-
Nephrotoxicity	-	-	-	-

CR, complete remission; Dfd, death from disease; GPS, Glasgow-prognostic score; NCI CTC, National Cancer Institute Common Toxicity Criteria; PD, progressive disease; PR, partial remission; PRRT, peptide-receptor-radionuclide-therapy; SD, stable disease; RECIST, Response evaluation criteria in solid tumors.
